# New 4-Thiazolidinones of Nicotinic Acid with 2-Amino-6-methylbenzothiazole and their Biological Activity

**DOI:** 10.3797/scipharm.1009-15

**Published:** 2010-10-24

**Authors:** Navin B. Patel, Faiyazalam M. Shaikh

**Affiliations:** Department of Chemistry, Veer Narmad South Gujarat University, Surat 395007, Gujarat, India

**Keywords:** Antimicrobial activity, Schiff bases, 4-Thiazolidinones, Nicotinic acid

## Abstract

The title compounds **6a–j**, 2-[(6-methyl-1,3-benzothiazol-2-yl)amino]-*N*-[2-(substituted phenyl/furan-2-yl)-4-oxo-1,3-thiazolidin-3-yl]nicotinamides, were prepared from 2-chloropyridine-3-carboxylic acid (**1**) and 2-amino-6-methyl-benzothiazole (**2**) by known methods. All the compounds have been established by IR, ^1^H NMR, ^13^C NMR and elemental analyses. The *in vitro* antimicrobial screening of the compounds were carried out against two Gram positive (*S. aureus*, *S. pyogenes*), two Gram negative (*E. coli*, *P. aeruginosa*) bacteria and three fungal species (*C. albicans*, *A. niger*, *A. clavatus*) using the broth microdilution method. Some of the compounds are comparable with standard drugs.

## Introduction

A large number of drugs and biologically relevant molecules contain heterocyclic systems. Often the presence of hetero atoms or groupings imparts preferential specificities in their biological responses. The chemistry and biological study of heterocyclic compounds has been interesting field for a long time due to medicinal and agricultural reasons. The number of heterocyclic derivatives containing nitrogen and sulfur atom possess broad spectrum of biological activities. One of the most important heterocycle in medicinal chemistry is pyridine with wide application including antimicrobial, anti-inflammatory, anti-HIV, antiplasmodial, anti-tubercular, antibacterial and anticonvulsant [1–7] activities, and has much other important biological significance.

The 4-thiazolidinone ring system comprises the broad spectrum for a number of biologically active compounds. In recent years, 4-thiazolidinones are the most extensively investigated class of compounds, which exhibit various biological activities, such as antimicrobial, anti-inflammatory, anti-HIV, anti-toxoplasma gondii and analgesic [8–12].

Looking towards literature, it was thought that incorporation of all these biologically active moieties might be result in better antimicrobial activity and therefore as the part of our continuous research in developing the new heterocycles containing nitrogen and sulfur atom and screening their microbial studies [13–15], herewith we have designed 4-thiazolidinones incorporated nicotinic acid with 2-amino-6-methylbenzothiazole and examined their antimicrobial activities.

## Results and Discussion

### Synthesis of compounds

2-Chloro pyridine-3-carboxylic acid **1** and 2-amino-6-methyl benzothiazole **2** in presence of anhydrous K_2_CO_3_ and Cu-bronze in DMF solvent on Ullmann condensation yielded 2-[(6-methyl-2-benzothiazolyl)amino]nicotinic acid (**3**). Further heating **3** with SOCl_2_ and subsequent reaction with hydrazine hydrate in chloroform formed **4** which on condensation with substituted aromatic aldehydes in DMF gave **5a–j**. Thaizolidinones **6a–j** were synthesized by refluxing **5a–j** and thioglycolic acid in dry 1,4-dioxane for 12–14 h using a Dean-Stark apparatus (Scheme 1). Purity of the compounds was checked by TLC using ethyl acetate: toluene (1:3) as a solvent system. Structures were characterized by spectral data (FT-IR, ^1^H-NMR and ^13^C-NMR).

### Investigations, Results and Discussion

The *in vitro* antibacterial and antifungal activities of the compounds are shown in Table 1. The MICs (μg/ml) were carried out by broth microdilution method as described by Rattan [17].

#### Antibacterial Activity

From the screening results (Table 1), it is evident that compound **1** displayed good to moderate activity against all bacteria (150–250 μg/ml). 2-Amino-6-methylbenzothiazole (**2**), compound **3** and hydrazide **4** exhibited moderate to poor activity against all bacteria.

The result shows that compounds **5a**, **5e**, **5j**, **6d**, **6g** and **6j** exhibited good activity (25–100 μg/ml) against *E. coli*; **5d**, **5j**, **6d**, **6i** and **6j** exhibited good activity (50–100 μg/ml) against *P. aeruginosa*; **5a**, **5e**, **5f**, **5h**, **5j**, **6d**, **6b** and **6j** showed good to very good activity (25–150 μg/ml) against *S. aureus*; whereas **5b**, **5h**, **5j**, **6c**, **6i** and **6j** showed good activity (62.5–100 μg/ml) against *S. pyogenes* compared with ampicillin. All other compounds showed moderate activity.

#### Antifungal Activity

From the results of the antifungal activity (Table 1), it is evident that compounds **1, 2, 3** and **4** showed good to moderate activity against *C. albicans*.

Results also show that Schiff bases and 4-thiazolidinones possessed good activity against *C. albicans* while moderate activity against *A. niger* and *A. clavatus.* Compounds **5a**, **5d**, **5g**, **5j**, **6b**, **6e**, **6f** and **6j** showed better activity (100–500 μg/ml) against *C. albicans* when compared with griseofulvin, while all compounds showed poor to moderate activity against *A. niger* and *A. clavatus.*

## Conclusion

Most of the compounds are comparable with ampicillin. Compounds bearing –Cl, –NO_2_ groups and furan nucleus are more active than the remaining compounds. Compounds **5a**, **5d**, **5g**, **5j**, **6b**, **6e**, **6f** and **6j** were found to be active against *C. albicans* but they found poor with other fungal species.

## Experimental

All chemicals were of analytical grade and used directly. Melting points of the synthesized compounds were determined by open tube capillary method and were uncorrected. The purity of the compounds was checked by TLC using Merck silica gel 60 F_254_. IR spectra were recorded on a Perkin-Elmer RX 1 FTIR spectrophotometer, using potassium bromide pellets; the frequencies are expressed in cm^−1^. The ^1^H NMR and ^13^C NMR spectra were recorded with a Bruker Avance II 400 NMR spectrometer, using TMS as an internal reference, with DMSO-d*_6_* as solvent. The chemical shifts are reported in parts per million (δ ppm). Elemental analyses were performed on Carlo Erba 1180 CHN analyzer. All the results of elemental analyses were in an acceptable error range.

2-Amino-6-methylbenzothiazole (**2**), 2-[(6-methyl-1,3-benzothiazol-2-yl)amino]nicotinic acid (**3**) and 2-[(6-methyl-1,3-benzothiazol-2-yl)amino]nicotinohydrazide (**4**) were prepared by reported procedures [15, 16].

### General procedure for syntheses of substituted *N’*-benzylidene/(2-furylmethylene)-2-[(6-methyl-1,3-benzothiazol-2-yl)amino]nicotinohydrazides (5a–j)

Benzaldehyde (0.012 mole, 1.272 g) and 3–4 drops of glacial acetic acid were added to a solution of **4** (0.01 mole, 3.0 g) in DMF (30 mL). The reaction mixture was refluxed for 5–6 h and monitored by TLC on silica gel using ethyl acetate:toluene (1:3). The reaction mass was cooled and poured onto crushed ice and thus the separated solid was isolated, washed with water and recrystallized from ethanol to give **5a**. Other derivatives **5b–j** were prepared by the same method.

#### *N*’-Benzylidene-2-[(6-methyl-1,3-benzothiazol-2-yl)amino]pyridine-3-carbohydrazide (**5a**)

White solid, yield: 52 %, mp: 180–182 °C. IR (KBr) ν cm^−1^: 3328 (NH), 1645 (amide-I), 1554 (amide-II), 1224 (Amide-III), 1616 (C=N of Schiff base), 2872, 2945 (CH_3_). ^1^H NMR (400 MHz, DMSO-d*_6_*, TMS): δ 1.25 (s, 3H, H-16), 5.84 (s, 1H, H-8), 6.81–8.58 (m, 11H, H-4,5,6,11,13,14,19,20,21,22,23), 8.84 (s, 1H, H-7), 9.34 (s, 1H, H-17); ^13^C NMR (100MHz, DMSO-d*_6_*, TMS): δ 22.1 (C-16), 112.6 (C-3), 118.3 (C-14), 119.8 (C-5), 122.4 (C-11), 127.0 (C-13), 127.9 (C-20,22), 129.9 (C-19,23), 130.9 (C-10), 132.1 (C-21), 134.6 (C-18), 136.9 (C-4), 138.4 (C-12), 143.3 (C-8), 148.2 (C-6), 151.3 (C-15), 161.5 (C-2), 162.1 (C-7), 170.5 (C-9). Anal. Calcd. for C_21_H_17_N_5_OS: C, 65.10; H, 4.43; N, 18.09. Found: C, 65.05; H, 4.38; N, 18.01.

#### *N*’-(2-Chlorobenzylidene)-2-[(6-methyl-1,3-benzothiazol-2-yl)amino]pyridine-3-carbohydrazide (**5b**)

White solid, yield: 49 %, mp: 168–169 °C. IR (KBr) *ν* cm^−1^: 3321 (NH), 1648 (amide-I), 1552 (amide-II), 1223 (amide-III), 1614 (C=N of Schiff base), 2869, 2942 (CH_3_). ^1^H NMR (400 MHz, DMSO-d*_6_*, TMS): 1.26 (s, 3H, H-16), 5.81 (s, 1H, H-8), 6.81–8.56 (m, 10H, H-4,5,6,11,13,14,19,20,21,22), 8.87 (s, 1H, H-7), 9.36 (s, 1H, H-17). ^13^C NMR (100MHz, DMSO-d*_6_*, TMS): δ 22.3 (C-16), 112.1 (C-3), 118.3 (C-14), 119.6 (C-5), 121.9 (C-11), 126.9 (C-13), 127.1 (C-20), 127.9 (C-19), 130.4 (C-22), 130.8 (C-10), 132.3 (C-21), 133.5 (C-23), 134.9 (C-18), 136.7 (C-4), 137.9 (C-12), 143.5 (C-8), 148.1 (C-6), 150.9 (C-15), 161.7 (C-2), 162.3 (C-7), 170.1 (C-9). Anal. Calcd. for C_21_H_16_ClN_5_OS: C, 59.85; H, 3.83; N, 16.63. Found: C, 59.78; H, 3.75; N, 16.56.

#### *N*’-(4-Chlorobenzylidene)-2-[(6-methyl-1,3-benzothiazol-2-yl)amino]pyridine-3-carbohydrazide (**5c**)

White solid, yield: 56 %, mp: 156–158 °C. IR (KBr) *ν* cm^−1^: 3319 (NH), 1642 (amide-I), 1552 (amide-II), 1223 (amide-III), 1619 (C=N of Schiff base), 2867, 2941 (CH_3_). ^1^H NMR (400 MHz, DMSO-d*_6_*, TMS): δ 1.24 (s, 3H, H-16), 5.82 (s, 1H, H-8), 6.79–8.53 (m, 10H, H-4,5,6,11,13,14,19,20,22,23), 8.86 (s, 1H, H-7), 9.38 (s, 1H, H-17). ^13^C NMR (100MHz, DMSO-d*_6_*, TMS): δ 1.25 (s, 3H, H-16), 5.84 (s, 1H, H-8), 6.81–8.58 (m, 11H, H-4,5,6,11,13,14,19,20,21,22,23), 8.84 (s, 1H, H-7), 9.34 (s, 1H, H-17); ^13^C NMR (100MHz, DMSO-d*_6_*, TMS): δ 22.5 (C-16), 112.8 (C-3), 118.1 (C-14), 119.9 (C-5), 122.1 (C-11), 126.4 (C-13), 128.4 (C-20,22), 130.3 (C-19,23), 131.0 (C-10), 133.9 (C-18), 135.7 (C-21), 137.1 (C-4), 138.3 (C-12), 144.1 (C-8), 147.9 (C-6), 151.1 (C-15), 161.9 (C-2), 163.5 (C-7), 170.4 (C-9). Anal. Calcd. for C_21_H_16_ClN_5_OS: C, 59.85; H, 3.83; N, 16.63. Found: C, 59.79; H, 3.77; N, 16.58.

#### 2-[(6-Methyl-1,3-benzothiazol-2-yl)amino]-*N*’-(2-nitrobenzylidene)pyridine-3-carbohydrazide (**5d**)

Yellow solid, yield: 58 %, mp: 188–190 °C. IR (KBr) *ν* cm^−1^: 3322 (NH), 1640 (amide-I), 1551 (amide-II), 1221 (amide-III), 1615 (C=N of Schiff base), 2872, 2940 (CH_3_), 1370, 1512 (NO_2_). ^1^H NMR (400 MHz, DMSO-d*_6_*, TMS): δ 1.23 (s, 3H, H-16), 5.81 (s, 1H, H-8), 6.82–8.56 (m, 10H, H-4,5,6,11,13,14,19,20,21,22), 8.82 (s, 1H, H-7), 9.37 (s, 1H, H-17). ^13^C NMR (100MHz, DMSO-d*_6_*, TMS): δ 22.7 (C-16), 112.7 (C-3), 118.3 (C-14), 119.6 (C-5), 121.9 (C-11), 123.8 (C-22), 126.9 (C-13), 129.8 (C-19), 130.5 (C-10), 132.4 (C-21), 133.9 (C-20), 133.1 (C-18), 137.1 (C-4), 137.9 (C-12), 143.4 (C-8), 147.2 (C-23), 147.4 (C-6), 150.9 (C-15), 161.8 (C-2), 162.0 (C-7), 170.1 (C-9). Anal. Calcd. for C_21_H_16_N_6_O_3_S: C, 58.32; H, 3.73; N, 19.44. Found: C, 58.26; H, 3.67; N, 19.38.

#### 2-[(6-Methyl-1,3-benzothiazol-2-yl)amino]-*N*’-(3-nitrobenzylidene)pyridine-3-carbohydrazide (**5e**)

Yellow solid, yield: 54 %, mp: 176–178 °C. IR (KBr) *ν* cm^−1^: 3321 (NH), 1641 (amide-I), 1552 (amide-II), 1226 (amide-III), 1617 (C=N of Schiff base), 2870, 2941 (CH_3_), 1369, 1511 (NO_2_). ^1^H NMR (400 MHz, DMSO-d*_6_*, TMS): δ 1.27 (s, 3H, H-16), 5.87 (s, 1H, H-8), 6.83–8.56 (m, 10H, H-4,5,6,11,13,14,19,20,21,23), 8.89 (s, 1H, H-7), 9.37 (s, 1H, H-17). ^13^C NMR (100MHz, DMSO-d*_6_*, TMS): δ 22.5 (C-16), 112.6 (C-3), 118.3 (C-14), 119.7 (C-5), 121.1 (C-11), 121.7 (C-23), 126.8 (C-13), 128.9 (C-20), 130.6 (C-10), 131.8 (C-21), 132.9 (C-19), 134.1 (C-18), 137.2 (C-4), 137.8 (C-12), 143.1 (C-8), 147.1 (C-22), 147.1 (C-6), 151.1 (C-15), 161.7 (C-2), 162.8 (C-7), 170.2 (C-9). Anal. Calcd. for C_21_H_16_N_6_O_3_S: C, 58.32; H, 3.73; N, 19.44. Found: C, 58.26; H, 3.67; N, 19.38.

#### *N*’-(4-Hydroxybenzylidene)-2-[(6-methyl-1,3-benzothiazol-2-yl)amino]pyridine-3-carbohydrazide (**5f**)

White solid, yield: 61 %, mp: 191–192 °C. IR (KBr) *ν* cm^−1^: 3322 (NH), 1644 (amide-I), 1548 (amide-II), 1223 (amide-III), 1618 (C=N of Schiff base), 2868, 2941 (CH_3_), 3502 (OH). ^1^H NMR (400 MHz, DMSO-d*_6_*, TMS): δ 1.26 (s, 3H, H-16), 5.31 (s, 1H, H-24), 5.85 (s, 1H, H-8), 6.82–8.54 (m, 10H, H-4,5,6,11,13,14,19,20,22,23), 8.79 (s, 1H, H-7), 9.31 (s, 1H, H-17). ^13^C NMR (100MHz, DMSO-d*_6_*, TMS): δ 1.25 (s, 3H, H-16), 5.84 (s, 1H, H-8), 6.81–8.58 (m, 11H, H-4,5,6,11,13,14,19,20,21,22,23), 8.84 (s, 1H, H-7), 9.34 (s, 1H, H-17); ^13^C NMR (100MHz, DMSO-d*_6_*, TMS): δ 22.6 (C-16), 112.4 (C-3), 117.9 (C-20,22), 118.3 (C-14), 119.8 (C-5), 122.3 (C-11), 126.9 (C-13), 130.1 (C-19,23), 130.9 (C-10), 132.9 (C-18), 136.9 (C-4), 138.1 (C-12), 143.2 (C-8), 147.9 (C-6), 151.3 (C-15), 161.6 (C-2), 158.1 (C-21), 163.3 (C-7), 170.2 (C-9). Anal. Calcd. for C_21_H_17_N_5_O_2_S: C, 62.51; H, 4.25; N, 17.37. Found: C, 62.45; H, 4.18; N, 17.30.

#### *N*’-(4-Methoxybenzylidene)-2-[(6-methyl-1,3-benzothiazol-2-yl)amino]pyridine-3-carbohydrazide (**5g**)

Brown solid, yield: 59 %, mp: 170–171 °C. IR (KBr) *ν* cm^−1^: 3321 (NH), 1643 (amide-I), 1555 (amide-II), 1228 (amide-III), 1615 (C=N of Schiff base), 2873, 2939 (CH_3_), 1039, 1179 (OCH_3_). ^1^H NMR (400 MHz, DMSO-d*_6_*, TMS): δ 1.28 (s, 3H, H-16), 3.88 (s, 1H, H-24), 5.81 (s, 1H, H-8), 6.86–8.58 (m, 10H, H-4,5,6,11,13,14,19,20,22,23), 8.86 (s, 1H, H-7), 9.34 (s, 1H, H-17). ^13^C NMR (100MHz, DMSO-d*_6_*, TMS): δ 21.9 (C-16), 55.1 (C-24), 113.1 (C-3), 115.9 (C-20,22), 118.3 (C-14), 119.7 (C-5), 121.9 (C-11), 127.1 (C-13), 129.8 (C-19,23), 131.0 (C-10), 131.9 (C-18), 136.9 (C-4), 137.1 (C-12), 142.9 (C-8), 148.0 (C-6), 151.1 (C-15), 160.8 (C-2), 162.0 (C-21), 163.1 (C-7), 170.0 (C-9). Anal. Calcd. for C_22_H_19_N_5_O_2_S: C, 63.29; H, 4.59; N, 16.79. Found: C, 63.23; H, 4.50; N, 16.70.

#### *N*’-(4-Hydroxy-3-methoxybenzylidene)-2-[(6-methyl-1,3-benzothiazol-2-yl)amino]pyridine-3-carbohydrazide (**5h**)

White solid, yield: 64 %, mp: 145–146 °C. IR (KBr) *ν* cm^−1^: 3323 (NH), 1645 (amide-I), 1554 (amide-II), 1221 (amide-III), 1616 (C=N of Schiff base), 2874, 2942 (CH_3_), 3507 (OH). ^1^H NMR (400 MHz, DMSO-d*_6_*, TMS): δ 1.26 (s, 3H, H-16), 3.86 (s, 3H, H-24), 5.38 (s, 1H, H-25), 5.80 (s, 1H, H-8), 6.83–8.58 (m, 9H, H-4,5,6,11,13,14,19,20,23), 8.87 (s, 1H, H-7), 9.36 (s, 1H, H-17). ^13^C NMR (100MHz, DMSO-d*_6_*, TMS): δ 22.2 (C-16), 55.9 (C-24), 110.9 (C-23), 112.7 (C-3), 116.7 (C-20), 118.3 (C-14), 119.2 (C-5), 121.9 (C-11), 122.4 (C-19), 127.0 (C-13), 130.9 (C-10), 132.1 (C-18), 137.4 (C-4), 138.1 (C-12), 143.1 (C-8), 147.8 (C-6), 149.1 (C-22), 150.9 (C-15), 152.8 (C-21), 161.2 (C-2), 163.5 (C-7), 170.2 (C-9). Anal. Calcd. for C_22_H_19_N_5_O_3_S: C, 60.95; H, 4.42; N, 16.17. Found: C, 60.88; H, 4.36; N, 16.10.

#### N’-(4-Hydroxy-3-methoxy-5-nitrobenzylidene)-2-[(6-methyl-1,3-benzothiazol-2-yl)amino]pyridine-3-carbohydrazide (**5i**)

Yellow solid, yield: 48 %, mp: 198–199 °C. IR (KBr) *ν* cm^−1^: 3324 (NH), 1647 (amide-I), 1552 (amide-II), 1225 (amide-III), 1618 (C=N of Schiff base), 2871, 2939 (CH_3_), 1037, 1181 (OCH_3_), 3503 (OH). ^1^H NMR (400 MHz, DMSO-d*_6_*, TMS): δ 1.26 (s, 3H, H-16), 3.87 (s, 3H, H-24), 5.32 (s, 1H, H-25), 5.84 (s, 1H, H-8), 6.82–8.56 (m, 8H, H-4,5,6,11,13,14,19,23), 8.87 (s, 1H, H-7), 9.37 (s, 1H, H-17). ^13^C NMR (100MHz, DMSO-d*_6_*, TMS): δ 22.5 (C-16), 55.7 (C-24), 112.6 (C-3), 117.4 (C-19), 118.0 (C-23), 118.3 (C-14), 119.7 (C-5), 121.9 (C-11), 127.0 (C-13), 129.1 (C-18), 130.6 (C-10), 136.7 (C-20), 137.4 (C-4), 138.2 (C-12), 140.8 (C-21), 143.3 (C-8), 147.7 (C-6), 150.5 (C-15), 151.7 (C-22), 161.5 (C-2), 163.1 (C-7), 170.5 (C-9). Anal. Calcd. for C_22_H_18_N_6_O_5_S: C, 55.22; H, 3.79; N, 17.57. Found: C, 55.16; H, 3.70; N, 17.50.

#### *N*’-(Furan-2-ylmethylidene)-2-[(6-methyl-1,3-benzothiazol-2-yl)amino]pyridine-3-carbohydrazide (**5j**)

Black solid, yield: 54 %, mp: 181–184 °C, IR (KBr) *ν* cm^−1^: 3320 (NH), 1646 (amide-I), 1553 (amide-II), 1223 (amide-III), 1617 (C=N of Schiff base), 2869, 2941 (CH_3_). ^1^H NMR (400 MHz, DMSO-d*_6_*, TMS): δ 1.27 (s, 3H, H-16), 5.81 (s, 1H, H-8), 6.86–8.52 (m, 9H, H-4,5,6,11,13,14,19,20,21), 8.86 (s, 1H, H-7), 9.38 (s, 1H, H-17). ^13^C NMR (100MHz, DMSO-d*_6_*, TMS): δ 22.7 (C-16), 112.6 (C-3), 113.3 (C-20), 116.9 (C-14), 118.1(C-19), 119.8 (C-5), 121.1 (C-11), 126.3 (C-13), 130.6 (C-10), 133.3 (C-8), 136.7 (C-4), 137.4 (C-12), 143.7 (C-21), 147.2 (C-6), 148.4 (C-18), 150.3 (C-15), 161.5 (C-2), 162.7 (C-7), 170.2 (C-9). Anal. Calcd. for C_19_H_15_N_5_O_2_S: C, 60.46; H, 4.01; N, 18.57. Found: C, 60.39; H, 3.94; N, 18.50.

### General procedure for syntheses of 2-[(6-methyl-1,3-benzothiazol-2-yl)amino]-*N*-[2-(substituted phenyl/furan-2-yl)-4-oxo-1,3-thiazolidin-3-yl]pyridine-3-carboxamides (6a–j)

A mixture of **5a** (0.01 mole, 3.87 g), thioglycolic acid (0.015 mole, 1.38 g) and a pinch of anhydrous ZnCl_2_ in dry 1,4-dioxane (30 mL) was refluxed for 12–14 h. The reaction was monitored by TLC on silica gel using ethyl acetate: toluene (1:3); was cooled and neutralized with 10% sodium bicarbonate solution. The solid product separated was filtered, washed with water and recrystallized from ethanol to give **6a**. Similarly, other 2-[(6-methyl-1,3-benzothiazol-2-yl)amino]-*N*-[2-(substituted phenyl/furan-2-yl)-4-oxo-1,3-thiazolidin-3-yl]pyridine-3-carboxamides **6b–j** have been prepared by the same method.

#### 2-[(6-Methyl-1,3-benzothiazol-2-yl)amino]-*N*-(4-oxo-2-phenyl-1,3-thiazolidin-3-yl)pyridine-3-carboxamide (**6a**)

White solid, yield: 57 %, mp: 184–186 °C. IR (KBr) *ν* cm^−1^: 3321 (NH), 1715 (C=O of 4-thiazolidinone), 1646 (amide-I), 1556 (amide-II), 1224 (amide-III), 2878, 2950 (CH_3_). ^1^H NMR (400 MHz, DMSO-d*_6_*, TMS): δ 1.24 (s, 3H, H-18), 3.63 (s, 2H, H-9), 6.21 (s, 1H, H-10), 6.91–8.57 (m, 11H, H-4,5,6,13,15,16,21,22,23,24,25), 8.91 (s, 1H, H-7), 9.36 (s, 1H, H-19). ^13^C NMR (100MHz, DMSO-d*_6_*, TMS): δ 21.1 (C-18), 35.9 (C-9), 57.7 (C-10), 111.6 (C-3), 114.1 (C-16), 118.5 (C-5), 121.1 (C-13), 125.9 (C-15), 127.1 (C-22,24), 128.1 (C-21,25), 129.9 (C-12), 134.4 (C-14), 137.0 (C-4), 138.1 (C-20), 147.4 (C-6), 149.1 (C-17), 127.9 (C-23), 163.5 (C-2), 163.1 (C-7), 169.3 (C-8), 171.2 (C-11). Anal. Calcd. for C_23_H_19_N_5_O_2_S_2_: C, 59.86; H, 4.15; N, 15.18. Found: C, 59.80; H, 4.08; N, 15.12.

#### *N*-[2-(2-Chlorophenyl)-4-oxo-1,3-thiazolidin-3-yl]-2-[(6-methyl-1,3-benzothiazol-2-yl)amino]pyridine-3-carboxamide (**6b**)

White solid, yield: 63 %, mp: 196–197 °C. IR (KBr) *ν* cm^−1^: 3322 (NH), 1718 (C=O of 4-thiazolidinone), 1644 (amide-I), 1555 (amide-II), 1225 (amide-III), 2875, 2948 (CH_3_), 755 (C-Cl). ^1^H NMR (400 MHz, DMSO-d*_6_*, TMS): δ 1.25 (s, 3H, H-18), 3.63 (s, 2H, H-9), 6.20 (s, 1H, H-10), 6.89–8.58 (m, 10H, H-4,5,6,13,15,16,21,22,23,24), 8.93 (s, 1H, H-7), 9.33 (s, 1H, H-19). ^13^C NMR (100MHz, DMSO-d*_6_*, TMS): δ 23.7 (C-18), 35.7 (C-9), 57.8 (C-10), 102.9 (C-20), 111.2 (C-3), 116.9 (C-16), 119.1 (C-5), 120.9 (C-13), 126.1 (C-15), 126.4 (C-22), 127.9 (C-24), 128.4 (C-23), 129.9 (C-12), 130.1 (C-21), 133.8 (C-25), 134.4 (C-14), 137.1 (C-4), 147.2 (C-6), 149.7 (C-17), 163.4 (C-7), 163.7 (C-2), 169.2 (C-8), 171.3 (C-11). Anal. Calcd. for C_23_H_18_ClN_5_O_2_S_2_: C, 55.75; H, 3.66; N, 14.14. Found: C, 55.70; H, 3.59; N, 14.06.

#### *N*-[2-(4-Chlorophenyl)-4-oxo-1,3-thiazolidin-3-yl]-2-[(6-methyl-1,3-benzothiazol-2-yl)amino]pyridine-3-carboxamide (**6c**)

White solid, yield: 60 %, mp: 209–210 °C. IR (KBr) *ν* cm^−1^: 3323 (NH), 1716 (C=O of 4-thiazolidinone), 1646 (amide-I), 1560 (amide-II), 1226 (amide-III), 2872, 2946 (CH_3_), 756 (C-Cl). ^1^H NMR (400 MHz, DMSO-d*_6_*, TMS): δ 1.23 (s, 3H, H-18), 3.65 (s, 2H, H-9), 6.26 (s, 1H, H-10), 6.92–8.56 (m, 10H, H-4,5,6,13,15,16,21,22,24,25), 8.91 (s, 1H, H-7), 9.34 (s, 1H, H-19). ^13^C NMR (100MHz, DMSO-d*_6_*, TMS): δ 23.4 (C-18), 35.4 (C-9), 57.6 (C-10), 111.4 (C-3), 116.9 (C-16), 118.4 (C-5), 121.1 (C-13), 125.9 (C-15), 127.6 (C-22,24), 129.2 (C-21,25), 129.9 (C-12), 132.1 (C-23), 134.0 (C-14), 135.1 (C-20), 137.0 (C-4), 147.3 (C-6), 150.1 (C-17), 163.2 (C-7), 163.8 (C-2), 169.7 (C-8), 171.0 (C-11). Anal. Calcd. for C_23_H_18_ClN_5_O_2_S_2_: C, 55.75; H, 3.66; N, 14.14. Found: C, 55.69; H, 3.61; N, 14.07.

#### 2-[(6-Methyl-1,3-benzothiazol-2-yl)amino]-*N*-[2-(2-nitrophenyl)-4-oxo-1,3-thiazolidin-3-yl]pyridine-3-carboxamide (**6d**)

Yellow solid, yield: 56 %, mp: 219–221 °C. IR (KBr) *ν* cm^−1^: 3319 (NH), 1718 (C=O of 4-thiazolidinone), 1644 (amide-I), 1554 (amide-II), 1224 (amide-III), 2871, 2945 (CH_3_), 1371, 1514 (NO_2_). ^1^H NMR (400 MHz, DMSO-d*_6_*, TMS): δ 1.25 (s, 3H, H-18), 3.62 (s, 2H, H-9), 6.21 (s, 1H, H-10), 6.87–8.57 (m, 10H, H-4,5,6,13,15,16,21,22,23,24), 8.92 (s, 1H, H-7), 9.35 (s, 1H, H-19). ^13^C NMR (100MHz, DMSO-d*_6_*, TMS): δ 23.6 (C-18), 35.6 (C-9), 57.1 (C-10), 111.3 (C-3), 116.5 (C-16), 118.7 (C-5), 120.9 (C-13), 124.1 (C-24), 125.9 (C-15), 127.3 (C-23), 128.9 (C-21), 129.9 (C-12), 132.6 (C-22), 133.5 (C-20), 134.4 (C-14), 137.1 (C-4), 147.3 (C-6), 148.6 (C-25), 149.8 (C-17), 163.1 (C-7), 163.6 (C-2), 169.4 (C-8), 171.1 (C-11). Anal. Calcd. for C_23_H_18_N_6_O_4_S_2_: C, 54.54; H, 3.58; N, 16.60. Found: C, 54.48; H, 3.50; N, 16.56.

#### 2-[(6-Methyl-1,3-benzothiazol-2-yl)amino]-*N*-[2-(3-nitrophenyl)-4-oxo-1,3-thiazolidin-3-yl]pyridine-3-carboxamide (**6e**)

Yellow solid, yield: 52 %, mp: 213–214 °C. IR (KBr) *ν* cm^−1^: 3318 (NH), 1714 (C=O of 4-thiazolidinone), 1648 (amide-I), 1560 (amide-II), 1227 (amide-III), 2869, 2946 (CH_3_), 1365, 1510 (NO_2_). ^1^H NMR (400 MHz, DMSO-d*_6_*, TMS): δ 1.21 (s, 3H, H-18), 3.60 (s, 2H, H-9), 6.24 (s, 1H, H-10), 6.96–8.59 (m, 10H, H-4,5,6,13,15,16,21,22,23,25), 8.90 (s, 1H, H-7), 9.34 (s, 1H, H-19). ^13^C NMR (100MHz, DMSO-d*_6_*, TMS): δ 23.4 (C-18), 35.7 (C-9), 57.1 (C-10), 111.3 (C-3), 116.5 (C-16), 119.1 (C-5), 121.2 (C-13), 122.1 (C-23), 123.6 (C-22), 125.2 (C-25), 126.1 (C-15), 129.7 (C-12), 133.1 (C-21), 134.0 (C-14), 137.1 (C-4), 140.0 (C-20), 146.4 (C-24), 147.9 (C-6), 149.7 (C-17), 163.1 (C-7), 163.4 (C-2), 169.2 (C-8), 171.3 (C-11). Anal. Calcd. for C_23_H_18_N_6_O_4_S_2_: C, 54.54; H, 3.58; N, 16.60. Found: C, 54.47; H, 3.51; N, 16.55.

#### *N*-[2-(4-Hydroxyphenyl)-4-oxo-1,3-thiazolidin-3-yl]-2-[(6-methyl-1,3-benzothiazol-2-yl)amino]pyridine-3-carboxamide (**6f**)

Brown solid, yield: 57 %, mp: 236–238 °C. IR (KBr) *ν* cm^−1^: 3320 (NH), 1717 (C=O of 4-thiazolidinone), 1646 (Aamide-I), 1556 (amide-II), 1228 (amide-III), 2872, 2942 (CH_3_), 3506 (OH). ^1^H NMR (400 MHz, DMSO-d*_6_*, TMS): δ 1.24 (s, 3H, H-18), 3.61 (s, 2H, H-9), 5.30 (s, 1H, H-26), 6.19 (s, 1H, H-10), 6.86–8.52 (m, 10H, H-4,5,6,13,15,16,21,22,24,25), 8.90 (s, 1H, H-7), 9.36 (s, 1H, H-19). ^13^C NMR (100MHz, DMSO-d*_6_*, TMS): δ 23.2 (C-18), 35.5 (C-9), 57.3 (C-10), 111.3 (C-3), 114.7 (C-16), 115.6 (C-22,24), 118.6 (C-5), 121.0 (C-13), 125.9 (C-15), 129.8 (C-12), 130.3 (C-21,25), 131.6 (C-20), 134.3 (C-14), 136.9 (C-4), 147.8 (C-6), 149.7 (C-17), 156.8 (C-23), 163.1 (C-2), 163.7 (C-7), 169.4 (C-8), 171.4 (C-11). Anal. Calcd. for C_23_H_19_N_5_O_3_S_2_: C, 57.85; H, 4.01; N, 14.68. Found: C, 57.78; H, 3.94; N, 14.60.

#### *N*-[2-(4-Methoxyphenyl)-4-oxo-1,3-thiazolidin-3-yl]-2-[(6-methyl-1,3-benzothiazol-2-yl)amino]pyridine-3-carboxamide (**6g**)

Brown solid, yield: 51 %, mp: 249–251 °C. IR (KBr) *ν* cm^−1^: 3321 (NH), 1719 (C=O of 4-thiazolidinone), 1648 (amide-I), 1552 (amide-II), 1227 (amide-III), 2873, 2947 (CH_3_), 1036, 1189 (OCH_3_). ^1^H NMR (400 MHz, DMSO-d*_6_*, TMS): δ 1.24 (s, 3H, H-18), 3.64 (s, 2H, H-9), 3.82 (s, 3H, H-26), 6.23 (s, 1H, H-10), 6.93–8.55 (m, 10H, H-4,5,6,13,15,16,21,22,24,25), 8.92 (s, 1H, H-7), 9.35 (s, 1H, H-19). ^13^C NMR (100MHz, DMSO-d*_6_*, TMS): δ 21.7 (C-18), 35.5 (C-9), 55.2 (C-26), 59.6 (C-10), 111.9 (C-3), 112.6 (C-22,24), 114.5 (C-16), 118.7 (C-5), 120.9 (C-13), 125.8 (C-15), 128.9 (C-21,25), 129.8 (C-12), 130.9 (C-20), 134.4 (C-14), 136.8 (C-4), 148.1 (C-6), 149.3 (C-17), 157.9 (C-23), 163.3 (C-2), 164.6 (C-7), 169.4 (C-8), 171.5 (C-11). Anal. Calcd. for C_24_H_21_N_5_O_3_S_2_: C, 58.64; H, 4.34; N, 14.26. Found: C, 58.58; H, 4.29; N, 14.18.

#### *N*-[2-(4-Hydroxy-3-methoxyphenyl)-4-oxo-1,3-thiazolidin-3-yl]-2-[(6-methyl-1,3-benzothiazol-2-yl)amino]pyridine-3-carboxamide (**6h**)

White solid, yield: 53 %, mp: 225–226 °C. IR (KBr) *ν* cm^−1^: 3318 (NH), 1718 (C=O of 4-thiazolidinone), 1647 (amide-I), 1556 (amide-II), 1224 (amide-III), 2876, 2944 (CH_3_), 1034, 1191 (OCH_3_), 3510 (OH). ^1^H NMR (400 MHz, DMSO-d*_6_*, TMS): δ 1.22 (s, 3H, H-18), 3.63 (s, 2H, H-9), 3.87 (s, 1H, H-26), 5.32 (s, 1H, H-27), 6.21 (s, 1H, H-10), 6.90–8.58 (m, 9H, H-4,5,6,13,15,16,21,22,25), 8.94 (s, 1H, H-7), 9.32 (s, 1H, H-19). ^13^C NMR (100MHz, DMSO-d*_6_*, TMS): δ 23.8 (C-18), 35.4 (C-9), 55.9 (C-26), 57.6 (C-10), 111.1 (C-3), 114.1 (C-25), 114.7 (C-16), 115.6 (C-22), 118.9 (C-5), 121.1 (C-13), 122.1 (C-21), 125.9 (C-15), 130.1 (C-12), 131.8 (C-20), 134.1 (C-14), 137.1 (C-4), 146.9 (C-24), 147.2 (C-23), 147.9 (C-6), 149.7 (C-17), 163.7 (C-2), 164.7 (C-7), 168.9 (C-8), 171.2 (C-11). Anal. Calcd. for C_24_H_21_N_5_O_4_S_2_: C, 56.79; H, 4.17; N, 13.81. Found: C, 56.71; H, 4.11; N, 13.73.

#### *N*-[2-(4-Hydroxy-3-methoxy-5-nitrophenyl)-4-oxo-1,3-thiazolidin-3-yl]-2-[(6-methyl-1,3-benzothiazol-2-yl)amino]pyridine-3-carboxamide (**6i**)

Yellow solid, yield: 59 %, mp: 256–257 °C. IR (KBr) *ν* cm^−1^: 3511 (OH), 3321 (NH), 1715 (C=O of 4-thiazolidinone), 1646 (Amide-I), 1561 (Amide-II), 1226 (Amide-III), 2877, 2947 (CH_3_), 1035, 1192 (OCH_3_). ^1^H NMR (400 MHz, DMSO-d*_6_*, TMS): δ 1.23 (s, 3H, H-18), 3.62 (s, 2H, H-9), 3.85 (s, 1H, H-26), 5.35 (s, 1H, H-27), 6.20 (s, 1H, H-10), 6.87–8.56 (m, 8H, H-4,5,6,13,15,16,21,25), 8.91 (s, 1H, H-7), 9.34 (s, 1H, H-19). ^13^C NMR (100MHz, DMSO-d*_6_*, TMS): δ 23.4 (C-18), 35.7 (C-9), 55.7 (C-26), 57.3 (C-10), 111.7 (C-3), 116.9 (C-16), 118.8 (C-5,21), 120.2 (C-25), 121.1 (C-13), 125.9 (C-15), 130.1 (C-12), 132.9 (C-20), 134.0 (C-14), 136.2 (C-23), 136.9 (C-4), 137.6 (C-22), 147.8 (C-6), 149.7 (C-17), 152.1 (C-24), 163.5 (C-7), 163.8 (C-2), 169.7 (C-8), 171.6 (C-11). Anal. Calcd. for C_24_H_20_N_6_O_6_S_2_: C, 52.17; H, 3.65; N, 15.22. Found: C, 52.10; H, 3.58; N, 15.15.

#### *N*-[2-(Furan-2-yl)-4-oxo-1,3-thiazolidin-3-yl]-2-[(6-methyl-1,3-benzothiazol-2-yl)amino]pyridine-3-carboxamide (**6j**)

Black solid, yield: 59 %, mp: 223–225 °C. IR (KBr) *ν* cm^−1^: 3419 (NH), 1715 (C=O of 4-thiazolidinone), 1645 (amide-I), 1558 (amide-II), 1225 (amide-III), 2874, 2948 (CH_3_). ^1^H NMR (400 MHz, DMSO-d*_6_*, TMS): δ 1.22 (s, 3H, H-18), 3.60 (s, 2H, H-9), 6.20 (s, 1H, H-10), 6.78–8.51 (m, 9H, H-4,5,6,13,15,16,21,22,23), 8.91 (s, 1H, H-7), 9.34 (s, 1H, H-19). ^13^C NMR (100MHz, DMSO-d*_6_*, TMS): δ 23.8 (C-18), 35.0 (C-9), 57.1 (C-10), 105.4 (C-22),109.7 (C-21), 111.2 (C-3), 116.5 (C-16), 118.9 (C-5), 121.1 (C-13), 125.9 (C-15), 130.1 (C-12), 134.0 (C-14), 137.0 (C-4), 141.5 (C-23), 147.3 (C-6), 149.7 (C-17), 151.1 (C-20), 163.1 (C-2), 163.8 (C-7), 169.1 (C-8), 170.9 (C-11). Anal. Calcd. for C_21_H_17_N_5_O_3_S_2_: C, 55.87; H, 3.80; N, 15.52. Found: C, 55.80; H, 3.72; N, 15.45.

### Experimental protocol for Antimicrobial activity

All the compounds were tested for their antibacterial and antifungal activity (MIC) *in vitro* by broth dilution method against two Gram positive *S. aureus* MTCC 96, *S. pyogenes* MTCC 442 and two Gram negative *E. coli* MTCC 443, *P. aeruginosa* MTCC 741 bacteria and three fungal species *C. albicans* MTCC 227, *A. niger* MTCC 282 and *A. clavatus* MTCC 1323. Ampicillin for antibacterial activity while griseofulvin for antifungal activity were used as a standard drug

The experimental protocol followed for antimicrobial activity was according to the same method as previously reported in literature [18, 19].

## Figures and Tables

**Fig. 1.**
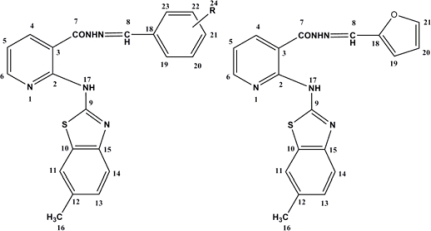
Numbering of substituted *N*’-benzylidene/(2-furylmethylene)-2-[(6-methyl-1,3-benzothiazol-2-yl)amino]nicotinohydrazides **5a–j**

**Fig. 2.**
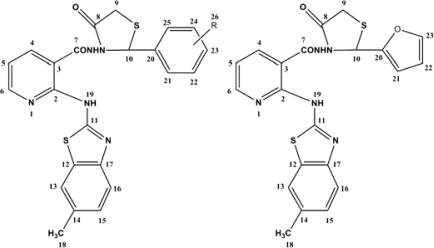
Numbering of 4-Thiazolidinones **6a–j**

**Sch. 1.**
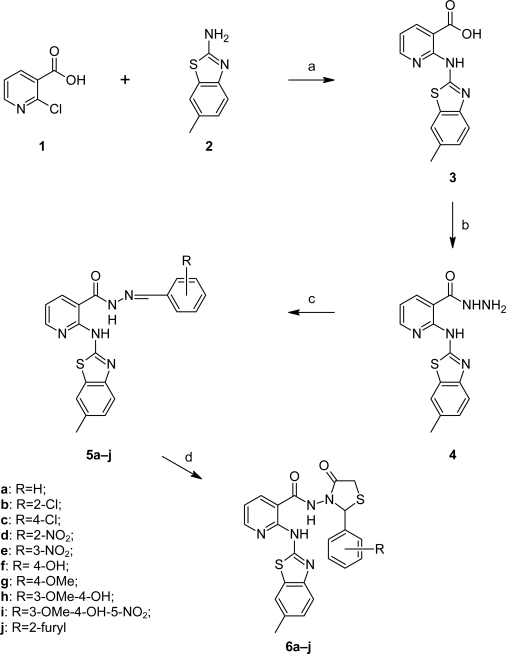
Reagents and conditions: (a) Ullmann condensation, Cu-Bronze, anhydrous K_2_CO_3_, DMF, reflux 5 h; (b) (i) SOCl_2_ and (ii) NH_2_NH_2_·H_2_O in CHCl_3_; (c) substituted aromatic aldehydes, DMF, reflux 5–6 h; (d) HSCH_2_COOH, anhydrous ZnCl_2_, 1,4-dioxane 12–14 h.

**Tab. 1.** Antibacterial and antifungal activities of **5a–j** and **6a–j**

**Comp.**	**Minimal bactericidal concentration μg/ml**				**Minimal fungicidal concentration μg/ml**		

	**Gram negative**		**Gram positive**				

	***E. coli***	***P. aerug.***	***S. aureus***	***S. pyogenus***	***C. albicans***	***A. niger***	***A. clavatus***
**1**	150	150	200	250	250	500	500
**2**	250	125	500	1000	1000	1000	1000
**3**	500	1000	500	1000	500	250	250
**4**	500	500	150	200	250	1000	1000
**5a**	100	500	150	200	100	500	500
**5b**	500	500	500	100	1000	1000	>1000
**5c**	500	500	1000	1000	1000	>1000	>1000
**5d**	250	62.5	500	500	250	1000	1000
**5e**	62.5	150	100	200	1000	500	500
**5f**	200	250	150	250	1000	1000	1000
**5g**	250	500	500	1000	150	500	500
**5h**	200	200	62.5	62.5	>1000	>1000	>1000
**5i**	500	500	500	500	>1000	500	>1000
**5j**	25	50	50	62.5	100	500	500
**6a**	250	250	500	500	1000	1000	1000
**6b**	500	500	150	500	150	1000	1000
**6c**	500	1000	1000	100	>1000	>1000	>1000
**6d**	50	100	100	150	>1000	1000	500
**6e**	250	250	500	500	500	>1000	>1000
**6f**	250	250	500	500	250	500	>1000
**6g**	62.5	200	500	500	1000	>1000	500
**6h**	200	500	500	500	1000	1000	1000
**6i**	500	100	1000	100	>1000	500	>1000
**6j**	100	62.5	25	100	500	1000	1000
**Ampicillin**	100	100	250	100	–	–	–
**Griseofulvin**	–	–	–	–	500	100	100
